# *PvMYB60* gene, a candidate for drought tolerance improvement in common bean in a climate change context

**DOI:** 10.1186/s40659-024-00528-8

**Published:** 2024-08-10

**Authors:** Vera Martínez-Barradas, Massimo Galbiati, Francisco Barco-Rubio, Dario Paolo, Carmen Espinoza, Eleonora Cominelli, Patricio Arce-Johnson

**Affiliations:** 1 Facultad de Ciencias Biológicas, Pontificia Universidad Católica de Chile, Santiago, Chile; 2 Facultad de Agronomía y Sistemas Naturales, Pontificia Universidad Católica de Chile, Santiago, Chile; 3 National Research Council, Institute of Agricultural Biology and Biotechnology (CNR-IBBA), Milan, Italy; 4 Instituto de Ciencias Biomédicas, Facultad de Ciencias de la Salud, Universidad Autónoma de Chile, Santiago, Chile; 5 Instituto de Ciencias Aplicadas, Facultad de Ingeniería, Universidad Autónoma de Chile, Santiago, Chile

**Keywords:** *MYB60*, Common bean, Drought stress, Drought tolerance, Transcription factor, Stomata opening

## Abstract

**Background:**

Common bean (*Phaseolus vulgaris*) is one of the main nutritional resources in the world, and a low environmental impact source of protein. However, the majority of its cultivation areas are affected by drought and this scenario is only expected to worsen with climate change. Stomatal closure is one of the most important plant responses to drought and the MYB60 transcription factor is among the key elements regulating stomatal aperture. If targeting and mutating the *MYB60* gene of common bean would be a valuable strategy to establish more drought-tolerant beans was therefore investigated.

**Results:**

The *MYB60* gene of common bean, with orthology to the Arabidopsis *AtMYB60* gene*,* was found to have conserved regions with MYB60 typical motifs and architecture. Stomata-specific expression of *PvMYB60* was further confirmed by q-RT PCR on organs containing stomata, and stomata-enriched leaf fractions. Further, function of *PvMYB60* in promoting stomata aperture was confirmed by complementing the defective phenotype of a previously described Arabidopsis *myb60-1* mutant.

**Conclusions:**

Our study finally points *PvMYB60* as a potential target for obtaining more drought-tolerant common beans in the present context of climate change which would further greatly contribute to food security particularly in drought-prone countries.

**Supplementary Information:**

The online version contains supplementary material available at 10.1186/s40659-024-00528-8.

## Introduction

In terms of direct human nutrition, common bean (*Phaseolus vulgaris)* is the most important legume globally [1]. Despite its origin in the Mesoamerican region, it is now cultivated worldwide [2]. The bean further plays a vital role in human diet by being one of the main nutritional sources in developing countries where it is also one of the most affordable sources of protein and nutrients [3]. The bean is also a low environmental impact source of protein when its nitrogen-fixing properties are considered[4, 5] and regarding growing global concerns about climate change, this legume could be a promising solution to the challenge of how to feed a growing global population with a crop with a reduced environmental impact [6, 7].

One of the impacts of climate change in agriculture is, however, the modification of precipitation regimes and water evaporation patterns [8]. These changes also pose a significant concern for common bean production. Approximately 60% of the areas where common beans are cultivated are exposed to moderate or extreme soil water deficit due to drought conditions [9, 10]. The cultivation of this crop heavily relies on rainfall; however, in areas with limited precipitation, irrigation becomes indispensable to maintain bean yields [11]. The impact of drought on common bean cultivation areas can result in losses ranging up to 100% [10]. Therefore, the need to improve drought tolerance in common beans is of great importance.

Plants cope with water limitation by optimizing water income, reducing water loss and optimizing cellular biochemistry with osmoprotectants, hormone signaling or better reactive oxygen species scavenging systems [12]. Reduction of water loss is therefore a promising strategy to develop more drought-tolerant plants [13, 14]. The structures that control water loss in plants are the stomata, which are composed of two guard cells that generate the stomatal pore when they are turgid. By changes in turgor, guard cells then control the pore aperture. The opening and closure of stomata depends, however, on water availability, external humidity, light, and also internal CO_2_ concentration [15]. Signals such as as second messengers, hormones and transcription factors further control the opening or closure of the stomata [16].

In particular the transcription factor MYB60 has attracted much attention for its role in controlling stomatal opening. MYB60 is a member of the MYB family [17] belonging to the R2R3 type of transcription factors because of the characteristics of its DNA binding motif [18]. MYB60 has been intensively studied in various plant species including *Arabidopsis thaliana* [19, 20]*, Vitis vinifera* [21, 22] and *Solanum lycopersicum* [23]*.* In these species MYB60 is specifically expressed in stomata, promotes stomatal opening, and its expression is diminished by abscisic acid (ABA), the main hormone involved in the plant’s drought response. Knocking-out the *MYB60* function reduces stomatal aperture by about 30% when compared to a control [20]. This is associated with decreased water loss in the plant and improved drought tolerance [20, 22, 23].

Unfortunately, there is so far no information avialable about the charactetistics of MYB60 in common bean. We therefore investigated whether a MYB60 conserved transcriptional regulation is also conserved in common bean and we hypothesized that the bean *MYB60* gene will have similar characteristics as previously found for *MYB60* genes identified in different other plant species. We first identified and characterized in our study the *Phaseolus vulgaris MYB60* gene, with the ultimate goal to establish the gene as a potential target in a strategy to improve, for example by mutation, the bean’s drought tolerance.

## Methods

### Plant material

The *P. vulgaris* BAT 93 genotype was kindly provided by the International Center for Tropical Agriculture (CIAT, Cali, Colombia) and used for all common bean analyses. Samples for gene expression analyses from different organs were collected from common bean seedlings or plants grown as previously described [24]. For the expression analyses on whole leaves and stomata enriched fractions, BAT 93 plants were grown in a phytotron at 25 °C under long-day conditions (14 h light at 26 °C, 10 h dark at 21 °C) at a relative humidity of 50%. *Arabidopsis thaliana* Columbia (Col-0) ecotype, *myb60-1* [20] and transgenic plants harbouring the *PvMYB60* coding sequence were grown under long-day conditions (14 h light/10 h dark at 100 μmol m^−2^ s^−1^) at 22 °C in a growth chamber either in Petri dishes or in soil. Transgenic lines were selected on Petri dishes, containing MS medium (Duchefa Biochemie, Haarlem, The Netherlands), 1% w/v sucrose, 0.8% w/v plant agar and hygromycin (15 μg/ml) [25].

### Phylogenetic analysis

A BLASTP with subgroup 1 proteins from the R2R3 MYB family and the previously characterized MYB60 protein sequences from *Solanum lycopersicum* and *Vitis vinifera* was done against the *P. vulgaris* annotated genome in the Phytozome platform (https://phytozome-next.jgi.doe.gov/). The 10 most similar protein sequences were retrieved from the NCBI Genebank (https://www.ncbi.nlm.nih.gov/). Sequences were aligned applying the MUSCLE algorithm, and a rooted phylogenetic tree was constructed with the maximum likelihood method with partial deletion for gap treating. The alignment and phylogenetic tree were done with the Mega software version X [26].

### Promoter analysis

The 2000 bp sequence upstream of the *PvMYB60* gene start codon was analysed with the PlantPan 4.0 software (http://plantpan.itps.ncku.edu.tw/) to identify specific domains, particularly DOF-binding sites [A/T]AAAG [27] and motifs associated to ABA repression in leaves and guard cells [28].

### Stomata enriched fractions purification

Five common bean leaflets of fully expanded leaves of similar size from 25 days seedlings were excised and their middle veins were discarded. Afterwards, they were whisked in a blender with ice-cold water and deionized water ice for one minute and filtered through a 210 µm nylon metallic net [22]. The whisking and filtering steps were repeated three times. Resulting epidermal fractions were immediately frozen in liquid nitrogen and processed for RNA extraction.

### Expression analyses by quantitative real time PCR

For RNA extraction, 100 mg of various organs were collected as previously described [24] and the total volume of the stomatal enriched fractions when corresponded, were ground with liquid nitrogen and then homogenised with 1 mL TRIzol reagent. In a next step, 200 µL of Phenol:Chlorophorm:Isoamyl alcohol (25:24:1) were added. The homogenised mixture was centrifuged (5 min, 13.000 g). Supernatant was recovered and mixed with 1 volume Phenol:Chlorophorm:Isoamyl alcohol (25:24:1) and centrifuged (10 min, 13.000 g). The supernatant was recovered for RNA precipitation with 1 volume of isopropanol. The pellet was dissolved with 400 µL TE buffer, and 400 µL 10 M LiCl were added. The mixture was incubated overnight at − 20 °C and then centrifuged (10 min, 13.000 g). The pellet was washed with 70% ethanol and resuspended in TE buffer. RNA was treated with TURBO DNase (Thermo Fischer Scientific) and afterwards retrotranscribed with MultiScribe reverse transcriptase (Thermo Fischer Scientific) according to manufacturer’s instructions. Twenty µL q-PCR reactions were carried out with three technical replicates for each sample applying a SYBR Green PCR Master Mix (Bio-Rad) in an iQ5 real-time PCR detection system (Bio-Rad, Hercules, CA, USA). The q-PCR amplification conditions were 95 °C for 3 min, followed by 40 cycles of 95 °C for 10 s, 60 °C for 30 s, followed by a melt cycle from 60 to 95 °C. The *PvMYB60* and *PvKAT1* (PHAVU_006G164300g) genes were amplified with oligonucleotide pairs qPvMYB60F- qPvMYB60R and PvKat1F-PvKat1R (0.3 μM, Additional file 1: Table S1), respectively. The *PvActin11* (Phvul.008G011000) gene [29], amplified with primers PvACT11F1 and PvACT11R1 (0.3 μM, Additional file 1: Table S1), was used for normalization of common bean samples. Relative gene expression analysis was calculated by the 2^−ΔΔCt^ method, using the Bio-Rad CFX Maestro software v. 4.0. Statistical analysis was performed with GraphPad Prism version 8.0.0 for Windows, GraphPad Software, San Diego, California USA. A One-way ANOVA and a Benjamini, Krieger and Yekutieli multiple-comparisons test were performed for tissue expression analysis, and a 2-way ANOVA and Tukey’s multiple comparison test were performed for stomata enriched purification fractions expression analysis. Primers used in the experiments are listed in Additional file 1: Table S1.

### Isolation of the PvMYB60 sequence, cloning and transformation of *myb60-1 Arabidopsis* plants

To test for functional orthology, the 1.3 kb synthesized *AtMYB60* promoter [27] was fused to the 1002 bp *PvMYB60* (Phvul.001G107600) coding sequence and the 240 bp 35S terminator sequence (Integrated DNA Technologies IA, USA). The resulting fragment was then cloned into the entry vector pDONR 207 by the BP reaction using the BP Clonase II enzyme mix (Thermo Fisher Scientific) according to manufacturer’s instructions. Subcloning to the PMDC162 destination vector [30] was performed using the LR clonase II enzyme mix (Thermo Fischer Scientific) according to the manufacturer’s instructions. The resulting binary vector was then applied to transform *Agrobacterium tumefaciens* GV3101 cells and the obtained Agrobacterium strain was used for the floral-dip transformation of Arabidopsis *myb60-1* plants [31]. The T1 transformed plants were finally selected on a hygromycin-containing medium [25]. Any putative complemented transformants were confirmed by PCR analysis with PvMYB60F and PvMYB60R primers (Additional file 1: Table S1). Six independent complementation lines were finally used for this study, hereafter referred as C1, C3, C5, C6, C7 and C8.

### Analysis of stomatal aperture

Fragments of epidermal peelings were obtained from dark-adapted Arabidopsis Col-0, *myb60-1,* and complementation lines. Fragments were incubated for 4 h, with the abaxial side facing down, in a stomatal opening solution (30 mM KCl, 10 mM MES-KOH, pH 6.5) in a growth chamber at 22 °C under light (PAR 70–80 μmol m^−2^ s^−1^). Epidermal peelings were photographed with a bright field microscope coupled to a digital camera. Images were processed using FIJI software [32] and width and length of stomatal pores were measured. Between 60 and 160 stomatal pores were measured for each Arabidopsis line. The data of the relation between width and length was then analysed with a one-way ANOVA and a Kruskal–Wallis multiple comparison test using the GraphPad Prism version 8.0.0 for Windows, GraphPad Software, San Diego, California USA.

## Results

### Phylogenetic analysis

**Fig. 1:**
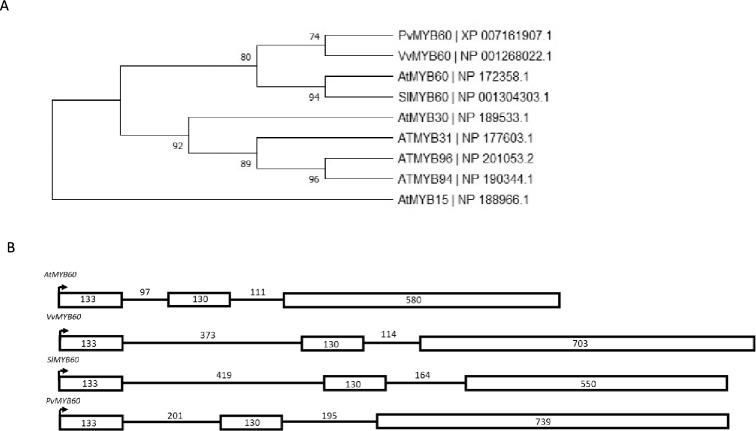
**A** Phylogenetic tree of PvMYB60, *Arabidopsis thaliana* subgroup 1 R2R3MYB transcription factors, and previously described MYB60 transcription factors. The tree was constructed using Maximum-Likelihood method. Numbers next to the branches represent the percentage of replicate trees in which the associated taxa clustered together in the bootstrap test (1000 replicates). **B** Comparison of the genetic structure of *PvMYB60*, *AtMYB60*, *VvMYB60*, and *SlMYB60*. Boxes represent exons, and lines introns. Arrows indicate transcription start site. Numbers indicate bp

To first identify the MYB60 homologue protein in the common bean genome, a BLASTP (Protein Blast) was performed using the aminoacidic sequences of all the R2R3 subgroup 1 transcription factors derived from *Arabidopsis thaliana* (AtMYB60, AtMYB96, AtMYB94, AtMYB31, and AtMYB30). Also included in Protein Blast were the previously characterized MYB60 sequences from *Vitis vinifera* and *Solanum lycopersicum* [21, 23]*.* The analysis showed that the XP 007161907.1 sequence from common bean was the most similar to all of the other blasted MYB60 sequences. A set up phylogenetic tree further confirmed this similarity. We, therefore, referred this sequence as *PvMYB60* (Fig. 1a). The genetic structure of *PvMYB60* was similar to the conserved structure of other *MYB60* genes consisting of three exons and two introns, with the first two exons having a highly conserved length (133 and 130 bp respectively), and a third variable exon of 739 bp in the case of *PvMYB60* (Fig. 1b).

**Fig. 2:**
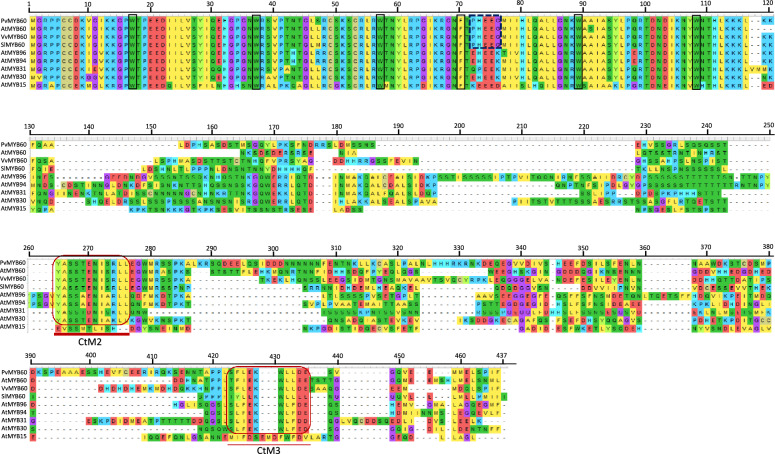
Alignment of PvMYB60 protein and its homologues. Aminoacid sequences of PvMYB60, AtMYB60, VvMYB60, SlMYB60 and Arabidopsis subgroup1 R2R3 MYB transcription factors were aligned with MUSCLE algorithm. Black boxes: regularly interspaced tryptophan and phenylalanine in R2R3 repeats. Red boxes: distinctive subgroup 1 R2R3 Carboxy-terminal motifs. Dark blue dotted box: PHEEG MYB60 signature. Dashes represent gaps in the alignment

In a further step, an in-detail analysis of the protein sequence was carried out. The PvMYB60 sequence shared the expected domains for a subgroup 1 R2R3 MYB transcription factor which is a DNA-binding motif characterized by an R1 motif with three regularly interspaced tryptophans, as well as an R3 motif with a phenylalanine and two tryptophans regularly interspaced [33]. Within the carboxy-terminal extreme, the C-terminal motif 2 (CtM2, YaSS^T^/_A_eNI^A^/S^R^/_K_L1) and CtM3 (SL[F/I]EKWLF[D/E]), motifs that characterize subgroup 1 R2R3 MYB transcription factors were further identified [33]. Between aminoacid residues 72–76, the MYB60 conserved signature PHEEG was also present in PvMYB60 sequence [21] (Fig. 2).

### Promoter analysis

By in silico analysis of the putative promoter region (2000 bp up-stream of the ATG) we found that this *PvMYB60* region had 19 DOF-binding sites in *cis* and 6 sites in *trans*. Interestingly, 12 of them grouped within 100 bp clusters, which have been previously shown to modulate gene expression in stomata [34]. Also, we found in the promoter region a total of 27 motifs associated with the negative regulation of *MYB60* by ABA [28] (Table 1 and Additional file 1: Fig. S1).

**Table 1:** Promoter analysis of *PvMYB60.* DOF binding sites [27], and ABA repression associated motifs unique to stomata and leaf expression [28]

	Position (+)	Position (−)
DOF binding site		
[A/T]AAAG	19	6
ABA repression associated motifs		
CAA[G/C]TTG	0	0
CCACT	2	0
CCAAC	0	0
CAACT	2	5
CACAT	4	4
GGTCC	0	0
TGCAA	2	5
GTCCC	1	1
GACCA	1	0

### *PvMYB60 *expression analyses

**Fig. 3:**
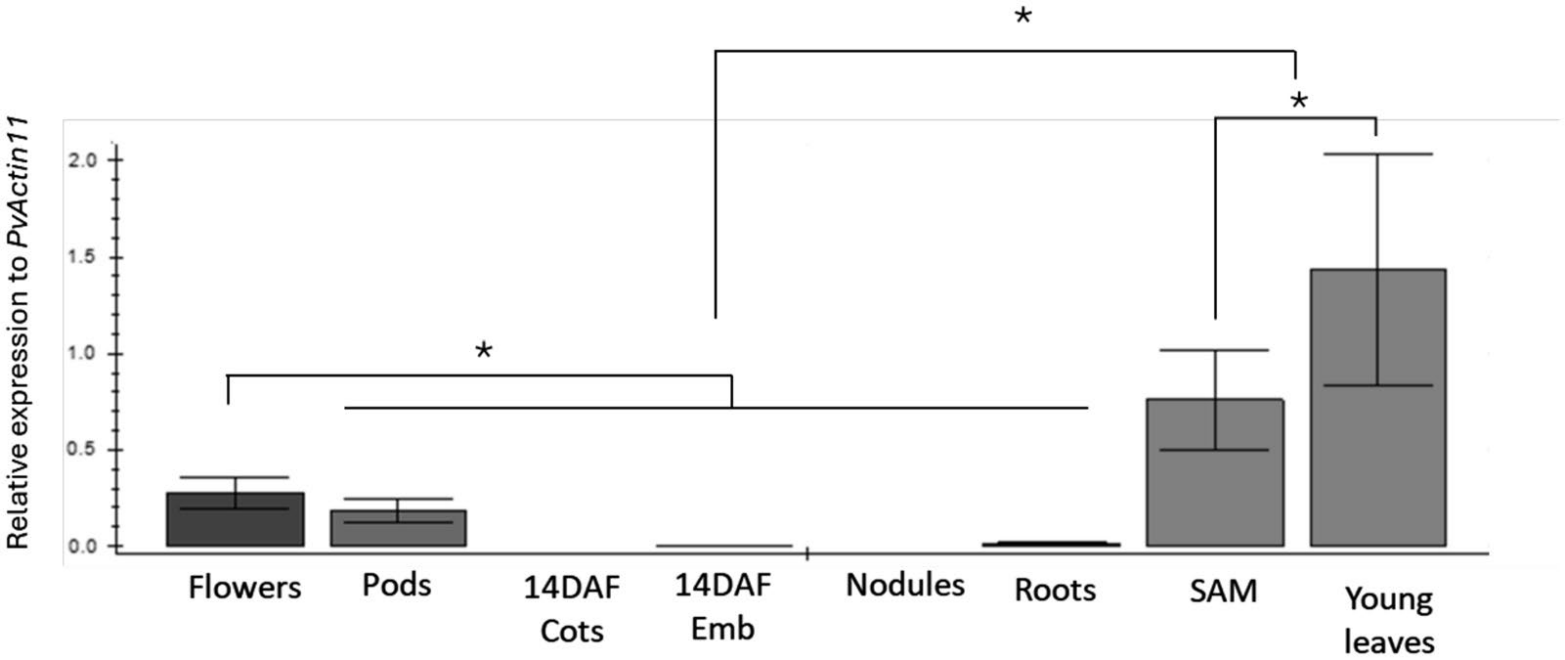
Expression of *PvMYB60* gene in different *P. vulgaris* organs. *PvMYB60* expression was quantified by qRT-PCR in flowers, pods, 14DAF cotyledons (14DAF cots), 14DAF embryos (14DAF Emb), nodules, roots, shoot apical meristems (SAM) and young leaves. *PvActin11* was used as a reference gene. Standard deviations are the result from three biological replicates. * indicates significant differences between tissues (*P* ≤ 0.05). One-way ANOVA and Benjamini, Krieger and Yekutieli multiple-comparisons test

**Fig. 4:**
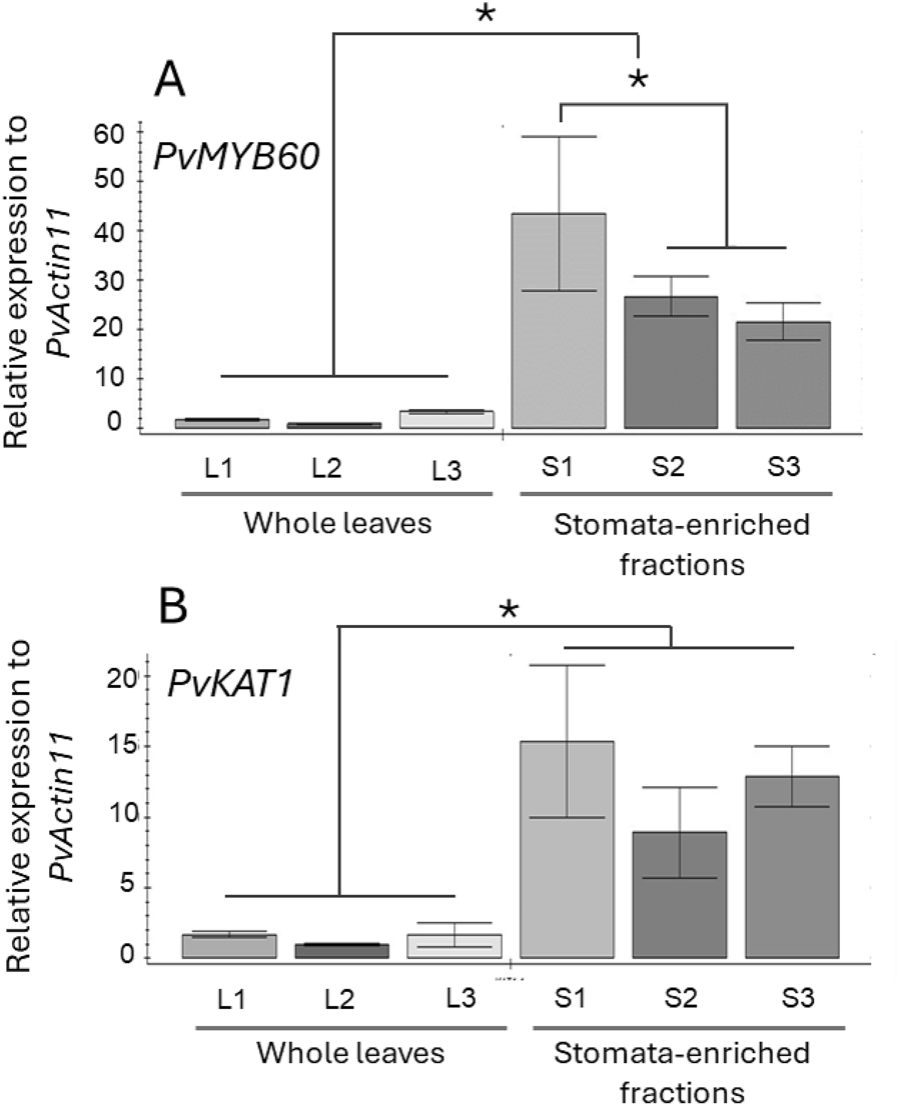
Expression of *PvMYB60* and *PvKAT1* in leaves and stomata-enriched fractions*.* **A** Expression of *PvMYB60* relative to *PvActin11* in three biological replicates for whole leaves (L) and stomata-enriched fractions (S). **B** Expression of *PvKAT1* relative to *PvActin11* in three biological replicates for whole leaves (L) and stomata-enriched fractions (S). Numbers in x axis stand for the corresponding replicate number. * indicates significant differences between samples (*P* ≤ 0.05). 2-way ANOVA and Tukey’s multiple-comparisons test

The whole organ expression results suggested *PvMYB60* to be expressed in tissues where stomata are present. To confirm this, *PvMYB60* expression was analysed in whole leaves and in stomata-enriched leaf fractions. The *PvKAT1*, identified as the common bean putative orthologue of the stomata-specific potassium channel *AtKAT1* gene [35], was used as a control. When we analysed the *PvMYB60* expression in different common bean organs, including reproductive and vegetative organs, and seeds at different developmental stages, the highest *PvMYB60* expression was detected in young leaves and shoot apical meristems, which were followed by flowers and pods. Fourteen days after flowering (DAF) cotyledons, 14 DAF embryos, nodules as well as roots had almost neglectable *PvMYB60* expression (Fig. 3). Since *PvMYB60* is expressed in leaves, we expected that expression is high in stomata-enriched fractions thereby following the same expression pattern as the putative K^+^ channel gene *PvKAT1* which codes for a guard cell-specific potassium channel [35]. We, therefore, purified stomata-enriched fractions from leaves and compared *PvMYB60* expression with expression of *PvKAT1* which is also highly similar to *AtKAT1*. *PvMYB60* and *PvKAT1* had the same expression pattern, with significantly higher expression in stomata-enriched fractions when compared to whole leaves (Fig. 4).

### *myb60-1: PvMYB60* mutant complementation test

**Fig. 5:**
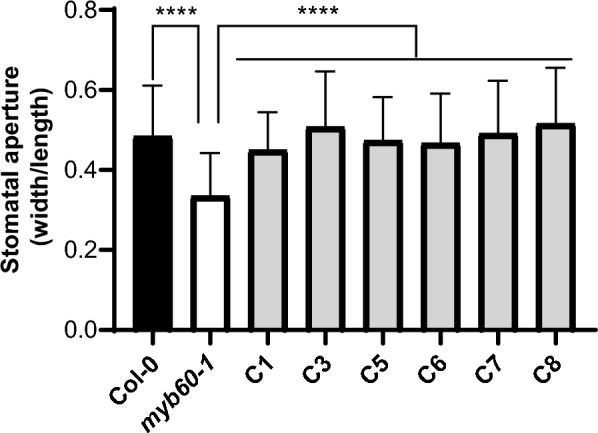
Stomatal aperture of the Arabidopsis *myb60-1*mutant complemented with the *PvMYB60* gene. Stomatal aperture is presented as the ratio between width and length measurements. A minimum of 60 stomata were measured for each line. **** indicate significant differences (*P* ≤ 0.0001) between different lines. One-way ANOVA and Kruskal–Wallis multiple comparison tests were performed. C1–C8 correspond to independent complementary Arabidopsis lines

*AtMYB60* is involved in stomatal aperture control. Arabidopsis *myb60-1* plants are further known to have a reduced stomatal aperture in comparison to wild-type (Col-0) plants [20]. To verify whether expression of the *PvMYB60* gene could complement the *myb60-1* phenotype, we measured the stomatal width and length of wild-type (Col-0) Arabidopsis plants and in *PvMYB60* complemented Arabidopsis mutant lines. We used the width/length ratio as a measure of stomatal aperture. The mean stomata width/length ratio was significantly (*P* ≤ 0.0001) higher for Col-0 (0.48), than for the *myb60-1* mutant plants (0.33)*.* For the six complementation lines it varied between 0.45 and 0.51, but with no significant difference (*P* ≤ 0.0001) to Col-0 control plants (Fig. 5 and Additional file 1: Fig. 2), showing that the *PvMYB60* gene is able to complement the *myb60-1* phenotypic alteration.

## Discussion

Given the enormous need to secure nutritious food under conditions of low soil water availability, the specific goal of developing drought-tolerant plants is crucial and should, therefore, be subject of intensive basic and also applied research in plant science [36]. Any improved drought tolerance in common beans has so far only been achieved by applying traditional breeding techniques, such as crossings with tolerant cultivars [11, 37, 38].

In an attempt to identify precisely future targets to breed beans more tolerant to soil water-deficit caused by drought conditions, we characterized the *MYB60* orthologue from common bean, a gene that was previously identified as involved in stomatal movement modulation in other species.

We found strong support for our original working hypothesis that *PvMYB60* gene has similar characteristics as previously found for other *MYB60* genes. The phylogenetic analysis here presented grouped the PvMYB60 transcription factor with the previously described MYB60 transcription factors from various other plant species (*A. thaliana, V. vinifera* and *S. lycopersicum*, and more closely related to the one from *V. vinifera*)*.* We further identified in our analysis three exons in *PvMYB60*, with the first two highly conserved regarding their length, and a third exon, with a variable length which was the longest among the considered *MYB60* genes. The high sequence conservation of the first two exons could be indicative of their coding role for the R2R3 DNA binding domain, as also noted for other MYB60 proteins in existing literature [21, 23]. An in-detail analysis of the protein sequences further found a PvMYB60 similarity to subgroup 1 R2R3 MYB transcription factors. The identified sequence thereby presents the conserved tryptophan and phenylalanine residues in the regularly interspaced positions of the DNA binding regions of R2R3 MYB transcription factors. However, the carboxy-terminal domain groups PvMYB60 within the subgroup 1 due to the presence of CtM2 and CtM3 signatures [33, 39]. At the amino acidic level, we also identified the specific signature consisting of PHEEG residues in positions 73–77, in further agreement with the MYB60 characterization previously described by Galbiati et al. [21].

When looking at the sequence of the putative *PvMYB60* promoter region (assumed to be within 2000 bp from the ATG), the presence of 4 clustered DOF-binding sites supports the hypothesis that the gene is regulated to allow stomata-specific expression, as it happens for its orthologues in Arabidopsis, grapevine, and tomato [21, 23]. The DOF-binding sites cluster in the closest position to the ATG of *PvMYB60* sites between − 125 and − 220 bp; interestingly, the minimal promoter of the Arabidopsis *MYB60* gene similarly has a DOF-binding site cluster in close proximity of the ATG—between − 147 and − 210 bp—which is crucial to its regulative role [27]. Unique sequence motifs associated to the absiscic acid (ABA) transcriptome of Arabidopsis guard cells have been described before [28]. Remarkably, the in silico analysis of the promoter of the *PvMYB60* gene detected 27 motifs associated with negative transcriptional regulation by ABA, both in *cis* and *trans* positions. Negative regulation of *MYB60* by ABA has been extensively reported, supporting the idea that this phytohormone is pivotal in the response to drought stress [20, 21, 23]*.* Future studies should therefore investigate whether this hormonal pathway also regulates the expression of *PvMYB60*, as the presence of such elements in its promoter seems to suggest. To sum up, considering the highly conserved mechanisms of DOF-regulation among plants particularly in the abiotic stress responses [40] and the identification of ABA-related elements in the *PvMYB60* promoter, we can hypothesize a conserved regulation of the expression of the *PvMYB60* gene and of its orthologues, that the perspective of further investigation—including in vivo promoter characterization—should clarify.

Experimental analysis proved that the *PvMYB60* gene expression is restricted to organs where stomata are present and also provided strong evidence for stomata-specific expression. We found the highest expression in young leaves and SAM, where its expression can be expected because stomata have already been developed in the new leaves near the apical meristems [41]. We also found expression in flowers and pods, which are non-foliar organs but bearing stomata [42, 43]. *PvMYB60* expression was not found in roots nor in cotyledons, embryo, or nodules which is in accordance with previously reported findings [20].

A significative finding of this work is the proof that expressing the coding sequence of *PvMYB60* complements the defective stomatal aperture of the Arabidopsis *myb60-1* mutant, a strong indication that MYB60 functional orthology is maintained between the model species Arabidopsis and common bean. Cominelli et al. [20] reported that *myb60-1* Arabidopsis mutants have a reduced stomatal aperture that results in reduced water loss and a drought-tolerant plant phenotype. We are therefore also evaluating the strategy to obtain *myb60* knock-out mutants in common beans, which might result in more drought-tolerant beans due to a reduced stomatal opening in bean plants. Application of genome editing (GE) with CRISPR-Cas9 might be a powerful future tool to award better stress tolerance in crops [44]. It was very recently proven how CRISPR-based GE can be used for functional chatacterization of MYB genes also in non-model species, for example in shedding light on the role regulation of flavonoid biosynthesis via *MYB45* in tartary buckwheat (*Fagopyrum tataricum*) and via *MYB134* and *MYB115* in poplar (Populus *spp.*) [45, 46]. However, to this date, no gene-edited plants have been described for common beans. Genetic transformation of common beans was successfully obtained via biolistics (most recently by Ferreira et al. [47]) or via *Agrobacterium tumefaciens* (most recently by [48]), however efficiencies remain low due to the fact common bean is recalcitrant to in vitro regeneration and that all methods show a strong genotype-dependency [49]. To overcome such difficulties, future attempts to edit *PvMYB60* could for instance rely the use of novel CRISPR-vectors, designed for the co-delivery via the same T-DNA of CRISPR-Cas9 cassettes and overexpression cassettes of transcriptional developmental regulators such as BABY BOOM/BBM, WUSCHEL/WUS or the GROWTH REGULATING FACTOR/GRF and GRF-INTERACTING FACTOR/GIF chimera (GRF4-GIF) that proved succesfull in increasing the speed and efficiency of regeneration in both monocots and dicots and produced fertile edited plants [50].

## Conclusions

This study functionally characterized the *PvMYB60* gene and allows to point at it as a promising candidate for the precision-breeding of drought-tolerant common bean cultivars. Future analyses should focus on the regulation of its expression and on the regulatory network downstream of it; such data, coupled with phenotype characterization of *myb60* mutants of common bean, whether identified via classical mutagenesis or novel methods of genome editing, would significantly expand the currently existing knowledge on *MYB60* function in drought tolerance also to common bean.

### Supplementary Information

**Additional file 1: Table S1**. Primer pairs used in qPCR and PCR. **Fig. S1**. DOF-binding sites in the putative promoter region of *PvMYB60*. DOF clusters (Galbiati et al. 2008) are underlined in red and numbered starting from the closest to the *PvMYB60* transcription start site. **Fig. S2**. Stomatal opening representative images. Three representative images from Col-0 and *myb60-1* lines are shown, indicated as A, B and C. For complemented lines, a representative image of stomatal opening corresponding to each treatment is shown. C1–C8 correspond to independent complementary Arabidopsis lines.

## Data Availability

The datasets used and/or analysed during the current study are available from the corresponding author on reasonable request.
