# Supplementary material for: PvMYB60 gene, a candidate for drought tolerance improvement in common bean in a climate change context
Source: Biol Res. 2024 Aug 10;57:52. doi: 10.1186/s40659-024-00528-8 (PMC11316432; doi:10.1186/s40659-024-00528-8)
Supplement: Supplementary file 1 — Additional file 1: Table S1. Primer pairs used in qPCR and PCR. Fig. S1. DOF-binding sites in the putative promoter region of PvMYB60. DOF clusters (Galbiati et al. 2008) are underlined in red and numbered starting from the closest to the PvMYB60 transcription start site. Fig. S2. Stomatal opening representative images. Three representative images from Col-0 and myb60-1 lines are shown, indicated as A, B and C. For complemented lines, a representative image of stomatal opening corresponding to each treatment is shown. C1–C8 correspond to independent complementary Arabidopsis lines. [file 40659_2024_528_MOESM1_ESM.docx]

**Supplementary Table 1.** Primer pairs used in qPCR and PCR.

| **Primer** | **Sequence** |
| --- | --- |
| qPvMYB60F | CAAGTGGAGGAGATGATGGAGTTA |
| qPvMYB60R | AGAAACCTCAACCCATGTGTAATG |
| PvACT11F1 | TGCATACGTTGGTGATGAGG |
| PvACT11R1 | AGCCTTGGGGTTAAGAGGAG |
| PvKat1F | CATCTGCCACATCATATACAGG |
| PvKat1R | CATCTGAAACTCCTTGGAAGAG |
| PvMYB60 F | ATGGGGAGGCCTCCT |
| PvMYB60 R | TTAGAAGATTGGAGATAACTCCATC |


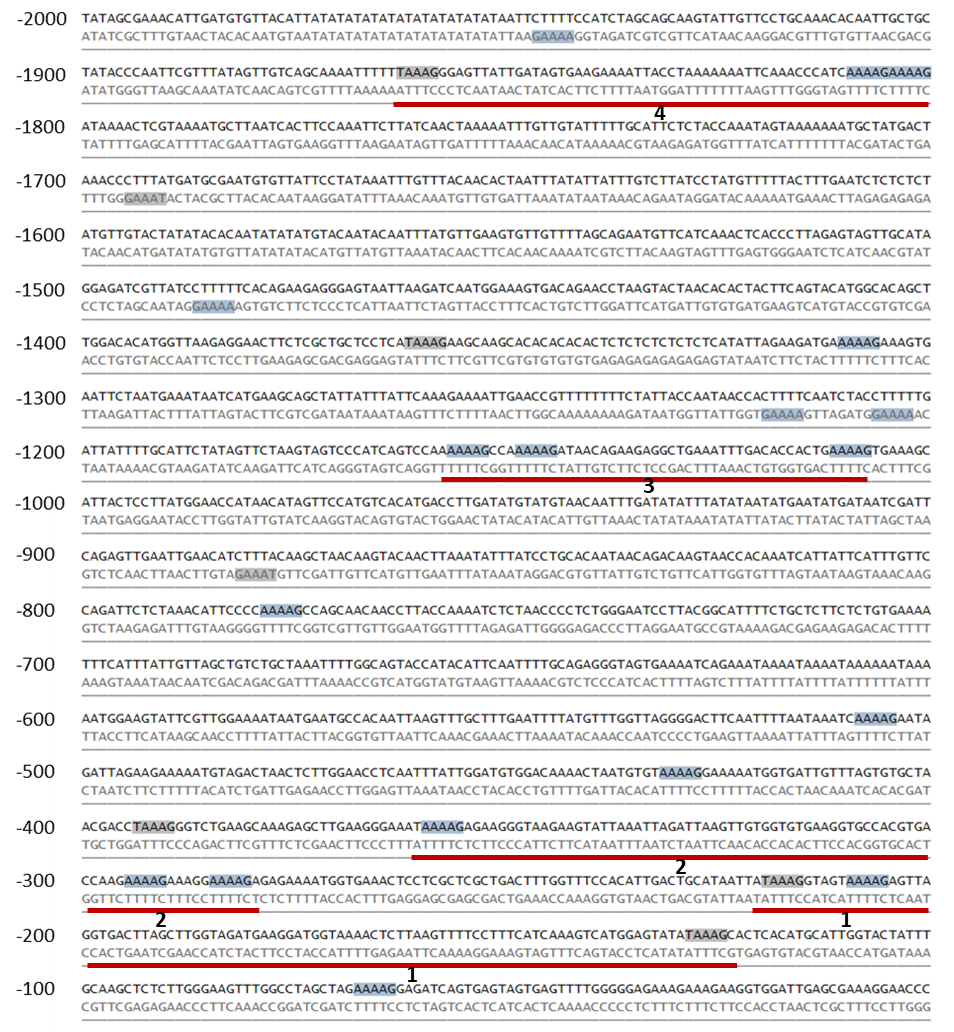


**Supplementary Figure 1**. DOF-binding sites in the putative promoter region of *PvMYB60.* DOF clusters (Galbiati et al., 2008) are underlined in red and numbered starting from the closest to the *PvMYB60* transcription start site.


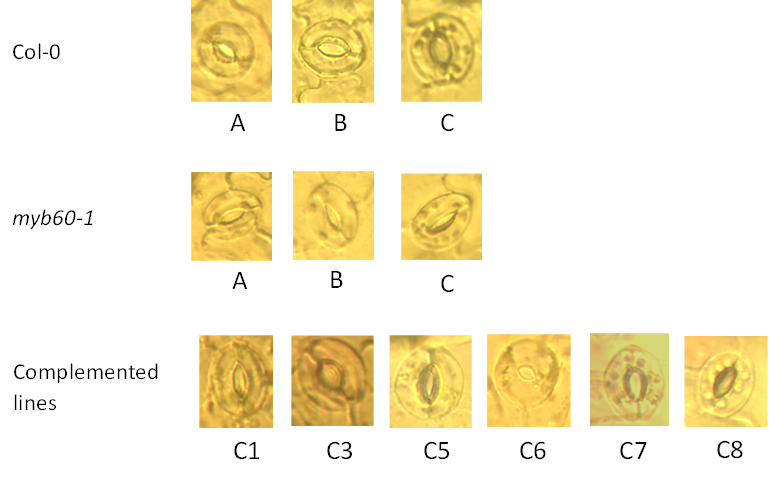


**Supplementary Figure 2.** Stomatal opening representative images. Three representative images from Col-0 and *myb60-1* lines are shown, indicated as A, B and C. For complemented lines, a representative image of stomatal opening corresponding to each treatment is shown. C1-C8 correspond to independent complementary Arabidopsis lines.
